# Comprehensive end‐to‐end dosimetry audit for stereotactic body radiotherapy in spine, lung, and soft tissue

**DOI:** 10.1002/acm2.70133

**Published:** 2025-06-05

**Authors:** Maddison Shaw, Andrew Alves, Jessica Lye, Joerg Lehmann, Fayz Kadeer, Sabeena Beveridge, Nicholas Hardcastle, Moshi Geso, Rhonda Brown

**Affiliations:** ^1^ Australian Clinical Dosimetry Service Australian Radiation Protection and Nuclear Safety Agency Melbourne Australia; ^2^ School of Health and Biomedical Sciences RMIT University Melbourne Australia; ^3^ Olivia Newton John Cancer Wellness Centre Melbourne Australia; ^4^ Department of Radiation Oncology Calvary Mater Newcastle Newcastle Australia; ^5^ School of Science RMIT University Melbourne Australia; ^6^ School of Mathematical and Physical Sciences University of Newcastle Newcastle Australia; ^7^ Institute of Medical Physics University of Sydney Sydney Australia; ^8^ Department of Physical Sciences Peter MacCallum Cancer Centre Melbourne Australia; ^9^ Sir Peter MacCallum Department of Oncology The University of Melbourne Melbourne Australia; ^10^ Centre for Medical Radiation Physics University of Wollongong Wollongong Australia

**Keywords:** dose to medium, dosimetry audit, multicentre study, QA, SABR, SBRT

## Abstract

**Purpose:**

To create and conduct a comprehensive onsite end‐to‐end dosimetry audit to assess treatment accuracy of spine, lung, and soft tissue Stereotactic Body Radiotherapy (SBRT) across Australian and New Zealand (ANZ) radiotherapy centers.

**Methods:**

The Australian Clinical Dosimetry Service (ACDS) anthropomorphic thorax phantom underwent a CT scan, planning, and treatment delivery according to local techniques at 128 facilities. Target volumes and dose constraints for spine, lung, and soft tissue were defined by the ACDS. Each plan was measured using Gafchromic EBT3 film and PTW 60019 microDiamond detector. A total of 782 plans were measured on 159 treatment machines of various classes and vendors. Audit results with the measured dose calculated as dose‐to‐medium, in medium (*D_m,m_*) or dose‐to‐scaled density water, in water (*D_w,w_*) were reported for all measurements, including those made in bone and lung equivalent materials.

**Results:**

The overall audit pass rate was 96% (271/281 plans) for the soft tissue case, 90% (215/238) for the spine, and 90% (236/263) for the lung. The average gamma pass rate for 5%/2mm criteria was 98.7% (soft tissue), 96.5% (spine), and 96.5% (lung). The average point dose difference was −1.0% (± 2.3%), 0.1% (± 3.8%), and −0.3% (± 3.2%) for the soft tissue, spine, and lung cases, respectively. The most common failure modes were in‐target dose differences (41.6%) and Image Guided Radiation Therapy (IGRT) mismatches (36.7%).

**Conclusions:**

High pass rates were seen for soft tissue, spine, and lung SBRT, indicating safe implementation of practice in the ANZ region. The modes of failure were assessed for suboptimal results, with the most frequent error due to IGRT mismatches, followed by dose differences in field, either underdosing or overdosing.

## 1 INTRODUCTION

Stereotactic Body Radiotherapy (SBRT) is an established modality in the treatment of early‐stage lung, prostate and liver cancers, and oligometastatic disease.^1^, ^2^, ^3^, ^4^, ^5^, ^6^, ^7^ SBRT target volumes present challenging conditions for dose calculation due to complexities from small field dosimetry, the presence of inhomogeneities, and proximity to critical organs. Common SBRT treatment sites, such as spine, lung, and soft tissue, each come with their complexities, and all are dependent on high‐quality image guidance and often motion management. Verification of treatment delivery is essential in ensuring patient safety and the realization of potential benefits of SBRT treatment approaches. Quality assurance is also of the utmost importance in the context of clinical trials, to ensure robust data is entered into studies of the efficacy of this style of treatment. End‐to‐end testing and independent external audits are recommended by SBRT guidelines worldwide.^8^, ^9^, ^10^, ^11^, ^12^

SBRT dosimetry audits have been performed for lung,^13^, ^14^, ^15^, ^16^, ^17^, ^18^ spine,^19^, ^20^, ^21^, ^22^ and liver^23^ treatment sites. These audits have been limited to a single anatomical site and were often designed to meet the credentialing needs of a specific clinical trial. Remote dosimetry audits^14^, ^19^, ^21^ remain popular due to lower costs and logistical overheads. On‐site audits offer the benefits of real‐time results, an overall discussion of SBRT clinical practices between facility staff and auditors, and error troubleshooting capability with facility staff. Previous onsite audits have involved a small number of treating facilities,^17^, ^18^, ^20^, ^22^ limiting the opportunity for trend analysis across common radiotherapy equipment types. In addition, some of the audits employed target (tumor) sizes which were larger than what would be considered typical of SBRT.^14^, ^19^, ^21^ Some of the audits used homogenous phantoms without realistic patient anatomy,^20^ or in‐house constructed phantoms with readily available materials as surrogates for patient tissues.^19^, ^21^, ^23^ Others report using commercially available anthropomorphic phantoms with tissue equivalent materials,^14^, ^17^, ^22^ reporting dose to water (*D_w,w_*) regardless of the measurement media in the phantom. A gap exists for a wide‐ranging SBRT audit which includes multiple treatment sites and corrects for measurements in non‐water materials such as bone and lung.

This work discusses the design, implementation, and results of an end‐to‐end SBRT dosimetry audit by the Australian Clinical Dosimetry Service (ACDS) across Australian and New Zealand (ANZ) radiotherapy facilities over a period of 6 years. The aims of this work were to deliver a comprehensive onsite dosimetry audit that collectively verifies treatment delivery for a range of clinical SBRT cases in one visit, using an anatomically realistic tissue equivalent anthropomorphic phantom. The audit is the first to directly report dose‐to‐medium (*D_m,m_*) or dose‐to‐scaled density water (*D_w,w_*) for measurements conducted in tissue equivalent materials, including bone and lung.^24^, ^25^ The ACDS SBRT audit allowed treatment verification in simple cases, plans with high modulation, and complex dose calculations in regions of spine and lung inhomogeneities. The wide range of clinical SBRT coverage could then be used to meet SBRT clinical use guidelines, and in credentialing in a number of national and international SBRT clinical trials.

## 2 MATERIALS AND METHODS

### 2.1 General audit procedures

**FIGURE 1:**
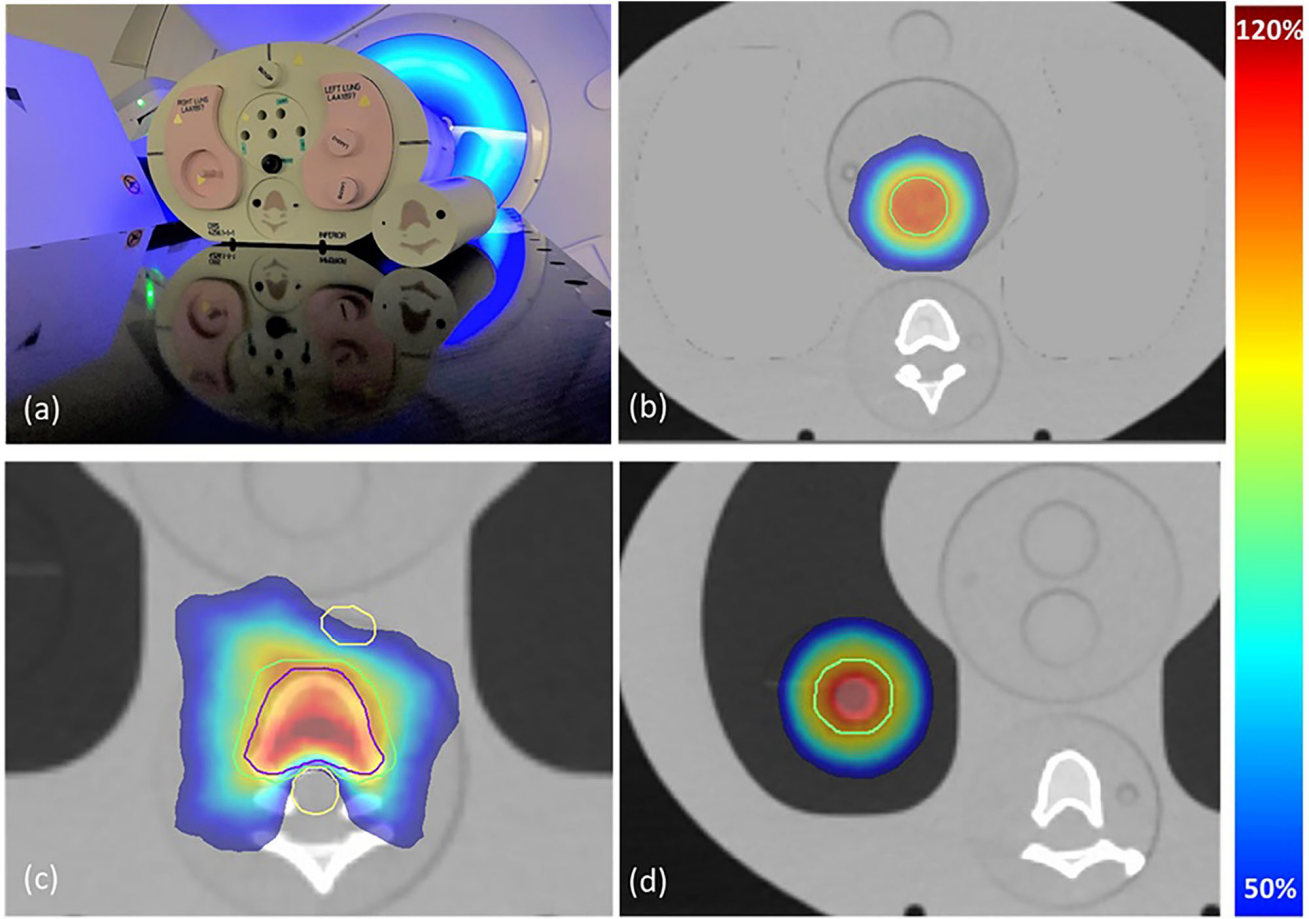
(a) ACDS SBRT anthropomorphic thorax phantom, (b) transverse view of soft tissue, (c) Spine, and (d) Lung plan and structures.

As discussed by Kron et al.^26^ and Lehmann et al.,^27^ a Level III dosimetry audit typically uses an anthropomorphic phantom and tests all aspects of the radiotherapy treatment chain, including imaging, dose calculation, and treatment. The ACDS Level III dosimetry audit for end‐to‐end testing previously covered 3D Conformal Radiotherapy (3DCRT), Intensity Modulated Radiotherapy (IMRT), and Volumetric Modulated Arc Therapy (VMAT).^27^, ^28^, ^29^ The SBRT modality was added to the Level III audit in 2018. The audit was performed onsite at 128 radiotherapy facilities across Australia and New Zealand, noting some facilities participated multiple times. The audit was performed using a customized anthropomorphic thorax phantom (Figure 1a, CIRS Inc.; Norfolk, VA, USA). The phantom comprised a water equivalent plastic “body”, inhale lung regions with a spherical 2 cm lung tumor in the right lung, and an anatomically correct spine composed of cortical and trabecular bone equivalent materials. Two similar bespoke phantoms were constructed by CIRS and used in audit operations. The phantoms had minor differences in their construction, particularly in the anthropomorphic spine, and were not interchangeable. The phantom was mailed to participating radiation oncology centers where it underwent a CT scan, treatment planning, and quality assurance checks according to the facility SBRT protocols. For planning purposes, planning target volumes (PTV) and organs at risk (OAR) were provided by the ACDS in a virtual dataset, which was fused with the facility CT scan. Plans were generated by the facility according to ACDS‐prescribed protocols. The beam energy and delivery modality were chosen by the facility as relevant to their local clinical practice. Submission of multiple plans per case was allowed. Audit dose measurements were performed onsite by ACDS representatives during treatment delivery. All aspects of treatment delivery, including image‐guided radiotherapy (IGRT), were completed by facility Radiation Therapists according to local clinical protocols. For plans to be accepted for the audit, facility staff must have deemed the plan to be clinically suitable for delivery, including passing facility patient‐specific quality assurance (PSQA) procedures.

The data collected for this analysis was completed during the initial go‐live period and as the audit progressed into routine operations. Results from 159 audits completed between April 2018 and April 2024 were included in this manuscript. The SBRT dosimetry audit continues to be performed routinely by the ACDS, supporting the safe treatment of patients in the ANZ region.

### 2.2 Audit cases

The audit involved three treatment cases replicating the most common tumor sites treated clinically with SBRT: soft tissue, spine, and lung.^30^, ^31^, ^32^, ^33^, ^34^ The soft tissue case was designed to replicate simple spherical targets commonly treated with SBRT, such as liver, prostate, and nodal volumes. A 2.5 cm diameter virtual target cylindrical PTV was defined in the center of the mediastinum of the thorax phantom (Figure 1b). Dose delivery for the soft tissue case was for a simple target volume without any adjacent OARs and without the presence of inhomogeneities in the calculation. For dose calculation, the lung regions were overridden to the density of the phantom plastic water body material to simulate a generic soft tissue abdominal treatment. The plan was then delivered with plastic water equivalent material in place of the lungs. The spine remained in place for IGRT purposes and was sufficiently far from the target that inhomogeneity modeling was not a significant contributor to the soft tissue case. The prescription dose for the soft tissue target was 45 Gy in 3 fractions, adapted from the CORE trial protocol for Liver SBRT.^35^ The prescription isodose was required to cover ≥95% of the PTV, with a maximum dose between 110% and 140% of the prescribed dose. The soft tissue PTV was not adjacent to any critical structures, simplifying the complexity of treatment delivery with minimal organ at risk constraints required in plans for this case. PTV Conformity Indices at 100% and 50% of the prescription dose (CI 100% and CI 50%) were required to be within 1.2 and 5.0, respectively. The CI values were based on the TROG SAFRON protocol,^36^ which were adapted from the ROSEL working party data.^37^

The spine clinical target volume (CTV) was defined as a single vertebral body, with a 2 mm margin applied for the Spine PTV. The Spine PTV excluded the Spinal Cord planning risk volume (PRV), resulting in a subtle wrap‐around target volume, with a surrogate esophagus OAR structure at the anterior (Figure 1c). The spine case was designed to require the most modulated delivery and the most difficult to achieve the planning constraints, due to the proximity of the target volume to the spinal cord and esophagus. The prescription dose for the spine PTV was 24 Gy in 2 fractions, adapted from the SC24 trial protocol for spine SBRT.^38^ The prescription isodose was required to cover ≥80%, but ideally ≥90% of the PTV, with a maximum dose between 110% and 140% of the prescribed dose. The maximum dose to 0.03 cc of the Spinal Cord PRV and Esophagus structures was 14 and 18 Gy, respectively. PTV Conformity Indices at 100% and 50% of the prescription dose were required to be within 1.2 and 5.0, respectively.

The lung case utilized a 2 cm diameter spherical target in the phantom's right lung, approximately 7 cm from midline. The first phase of the SBRT lung audit was designed to exclude motion management and employed a pseudo Internal Target Volume (ITV) approach, with application of a 1 cm isotropic margin to the visible tumor in the phantom (Figure 1d). The lung PTV was not adjacent to any critical structures, but included dose calculation in the presence of inhomogeneities. The prescription dose was 48 Gy in 4 fractions, adapted from the SAFFRON II trial protocol.^36^ The prescription dose was required to cover ≥98% of the PTV, with a maximum dose between 110% and 140% of the prescribed dose. The “normal lungs”, defined as the regions of the inhale lung minus the volume the PTV required, 5% of the total volume to receive <66%, and 12.4 Gy to less than 1000 cc. PTV Conformity Indices at 100% and 50% of the prescription dose were required to be within 1.2 and 5.0, respectively.

### 2.3 Dose detectors

The primary detector used in the audit was Gafchromic EBT3 film (Ashland; Bridgwater, NJ, USA) for both absolute dosimetry and 2D geometric accuracy. Film measurements were made in the center of the PTV in the transverse plane for the soft tissue and spine cases, and 3 mm anterior to the center of the PTV in the coronal plane for the lung case. The film was calibrated against a secondary standard Farmer‐type ionization chamber, using a normalized dose to water approach. Film calibration was periodically performed in‐house using the ARPANSA Elekta VersaHD Linac (Elekta AB; Stockholm, Sweden). This calibration process was chosen to eliminate the requirement of performing full film calibration whilst on site at the facility, reducing overall audit time. The calibration curve consisted of 12 irradiated films ranging from 0 to 20 Gy, using a 10 × 10 cm^2^ 6 MV field. Films were scanned using the EPSON Expression 12000XL flatbed scanner located onsite at ARPANSA. A dose‐dependent film darkening correction was applied to the calibration and audit films at the scanning process, to account for the difference in film darkening due to time between calibration and audit. During the initial phase of the audit, Film QA Pro software (FQAP) (Ashland; Bridgwater, NJ, USA) was used to generate absolute dose maps using triple channel optimization (RGB channels). Quality control investigations within the FQAP (pilot) method demonstrated inconsistencies with the dose map outputs. An improvement to the process introduced check films irradiated onsite at each audit. Two films were irradiated on the facility machine, with control doses (5 or 10 Gy, and 15 Gy), against a PTW 30013 Farmer‐type ionization chamber. These films were co‐scanned with the audit films and were used to linearly match the calibration. FQAP applied linear scaling without providing the scaling factor for inspection to check if it was within acceptable tolerance.

The FQAP method required multiple manual data entry steps and was open to changes to the configuration of the calibration method; thus, was considered particularly prone to manual data entry errors or unintended configuration changes. An in‐house film calibration software and method were subsequently developed to replace FQAP. The in‐house software utilized a Python program (v3.6, Python Software Foundation; Wilmington, DE, USA). Adopting the in‐house software resulted in an ability to maintain records of check film consistency and less requirement to double‐check film data processing, returning an overall improvement in user efficiency. Based on combined uncertainty in film uniformity, scanner repeatability, and the darkening correction, a quality control limit of 5% was set for linear scaling. Scans of the check films exceeded the control limit 2.5% of the time, and on each occasion, rescanning the films resolved the issue, indicating that scanner quality control is an important aspect in film dosimetry. Further details of the in‐house film process are discussed by Smyth et al.^39^ Based on the check film data and the exclusion of scans that failed the quality check film, we estimate the film dosimetric uncertainty is ± 2.5% (*k* = 1).

In the spine and lung cases, the film was measuring in patient tissue equivalent media (CIRS cortical bone, trabecular bone, inhale lung, and lung target material). Medium dependent corrections for measurement in synthetic patient equivalent materials were applied to the film as per the methodology described by Shaw et al.^24^, ^25^ The medium‐dependent corrections were tailored according to the dose reporting method of the algorithm used in the plan; dose‐to‐medium in medium (*D_m,m_*) or dose‐to‐water, in water (*D_w,w_*). The dose‐to‐water, in medium (*D_w,m_*) option available in Monte Carlo (MC) and AcurosXB (AXB) algorithms was not investigated as no plans were submitted using this reporting mode, following the guidance of the Global Harmonization Group for clinical trial QA.^40^

Complementary detectors used in the audit were PTW 60019 microDiamond detectors (PTW Freiburg; Germany) for point dose measurements. The microDiamond detector provided real‐time analysis of the measured audit plans whilst ACDS were onsite, and dosimetric comparisons with the film measurements. In the soft tissue case, the point dose was measured at the center of the PTV in the A/P and L/R planes, and 5 mm inferior to the film plane. For the spine case, two measurements were made in the PTV, superior and inferior to the film plane, and one measurement in the spinal cord region. The in‐PTV microDiamond measurement points for the spine were located in regions of trabecular bone. In the lung case, the microDiamond point was at the center of the PTV, with the film plane abutting anteriorly. The microDiamond measurement point for the lung case was located in the region of the lung target material. Corrections for measurements in cortical bone, trabecular bone, and lung target materials were applied to the microDiamond measurements, also tailored to the reporting mode of the plan algorithm.^24^, ^25^ For the soft tissue case, a water‐to‐tissue correction of 0.992 was applied to the microdiamond based on the recommendations of AAPM TG329.^41^ The microDiamond was cross‐calibrated against a secondary standard Farmer‐type ionization chamber.^42^ In all cases, the microDiamond detectors were placed into the phantom craniocaudally (along the superior/inferior axis). Additional corrections for detector orientation have been described by Shaw et al.^42^

### 2.4 Data analysis

Localization of film dose data to the treatment frame of reference was performed by matching physical cut‐outs on the films to the planning CT scan of the phantom provided by the facility. An in‐house MATLAB (Mathworks; Natick, MA, USA) code was developed with tools allowing the user to manually align film to the CT scan. The edges of the scanned audit film were overlaid with the facility planning CT scan and manually aligned by matching the cutouts in the film to physical pins within the phantom. The manual alignment was independently performed by two auditors with an agreement tolerance of <0.5 mm. The measured audit films were compared to the treatment plan using global gamma analysis (*γ*) and distance to agreement (DTA) between planned and measured isodose lines. For the 2D film data, the *k*_med_ factors were spatially dependent on the bone or lung regions and were applied in the in‐house program as described in Shaw et al.^24^, ^25^ Both the *D_w,w_* and *D_m,m_ k*_med_ factors were applied for all audit measurements. For the soft tissue and spine cases, a water‐to‐tissue correction of 0.992 was applied to the plastic water film regions based on the recommendations of AAPM TG329.^41^ When assessing audit outcomes, the k_med_ correction and water‐tissue corrections were tailored to the primary reporting mode of the plan algorithm. For benchmarking planning algorithms against each other, the *D_m,m_ k*_med_ factors were used.

Analysis of the overall audit results was performed using MATLAB and Microsoft Excel. Dose difference was defined as (planned‐measured)/measured (local) and (planned‐measured)/prescription dose per fraction (global). Dose differences are presented in box plots showing the median, upper, and lower quartiles for each data series. The statistical significance of uncorrected versus medium corrected results, as well as pilot FQAP film method versus in‐house film method, was evaluated using a two‐sample *t*‐test assuming equal variances, with the significance level *p* < 0.05.

### 2.5 Scoring criteria

The scoring criteria for the SBRT audit cases are detailed in Table 1. Thresholds were determined in discussion with an independent, multi‐disciplinary Clinical Advisory Committee after review of initial trial audit results. The case outcome is defined based on the global gamma criteria result. The passing ratio for global gamma criteria 5%/2mm, relative to the prescription dose, with <10% dose threshold, was used to define the audit outcome. The mean 1D DTA between planned and measured isodose lines in the horizontal and vertical profiles was assessed at the 70% isodose. For the spine case, the max DTA in the anterior‐posterior direction at the interface of the PTV/Spinal Cord was scored to assess the accuracy of the delivery with regard to the dose‐limiting Spinal Cord OAR. In the Lung case, only the anterior‐posterior DTA was assessed as the position of the film pin obstructed the 70% isodose in the left‐right direction. The local point dose variation for the microDiamond PTV point in each case was also reported. Upper limits were also placed on the DTA and point dose discrepancy metrics, which can return out‐of‐tolerance outcomes for an audit case regardless of gamma pass rate.

**TABLE 1:** Scoring criteria for SBRT audit.

Metric	Case	Criteria	Pass (optimal level)	Pass (action level)	Out of tolerance
Gamma Criteria	All	5%/2 mm	≥95%	<95% and≥90%	<90%
1D Profile DTA	Soft Tissue	Mean DTA @ 70% isodose (L‐R) and (A‐P)			>3.0mm
	Spine	Max DTA @ 70% isodose (A‐P) & Mean DTA @ 70% isodose (L‐R) at PTV‐Spinal Cord interface			>2.0 mm
	Lung	Mean DTA @ 70% isodose (A‐P)			>3.0mm
Point Dose Difference^a^	All	In‐PTV points			>8%

^a^Point dose difference = (Plan dose – Measured dose)/Measured dose.

The overall uncertainties in absolute dose and absolute position for the film measurements were ± 2.5% and ±0.4 mm, respectively. These figures are based on regions of interest and not per‐pixel analysis. The overall measurement uncertainty for the microDiamond measurements was 3.1%.^42^ For the point dose measurements, additional uncertainty exists due to the placement of interest points in the plan, which should be considered on an individual plan basis.

## 3 RESULTS

As summarized in Table 2, the audit was performed on a total of 159 treatment machines between April 2018 and April 2024, including conventional Varian and Elekta Linear Accelerators (Varian Medical Systems; Palo Alto, CA, USA and Elekta AB; Stockholm, Sweden), Varian Halcyon Linear Accelerators, and TomoTherapy and CyberKnife systems (Accuray; Sunnyvale, CA, USA). The audit was performed on some of the machines more than once. Plans were created for the audit cases using delivery techniques applicable to the facilities’ clinical practice. The majority of the plans included in the audit were delivered with VMAT, with IMRT, Dynamic Conformal Arc Therapy (DCAT), 3D 3DCRT, and cone‐based deliveries also measured (Table 2). A total of 782 plans were measured across three SBRT cases (soft tissue, spine, and lung). Plans were submitted for four photon beam energies: 6MV, 6MV FFF, 10 MV, and 10 MV FFF (Table 3). As summarized in Table 4, plans were submitted from seven Treatment Planning Systems (TPS); Eclipse (Varian Medical Systems; Palo Alto, CA, USA), Monaco (Elekta AB; Stockholm, Sweden), Pinnacle (Philips Radiation Oncology Systems; Milpitas, CA, USA), RayStation (RaySearch Laboratories; Stockholm, Sweden), iPlan (Brainlab AG; Munich, Germany), Precision and TomoTherapy Hi‐Art (Accuray; Sunnyvale, CA, USA). As detailed in Table 4, 11 dose calculation algorithms in different TPS implementations were submitted in audit plans, including Anisotropic Analytical Algorithm (AAA), AcurosXB (AXB), Monte Carlo/X‐Ray Voxel Monte Carlo (MC/XVMC), Collapsed Cone Convolution (Pinnacle CCC), Collapsed Cone (Raystation RS_CC), Adaptive Convolve (AC), and Ray Trace.

**TABLE 2:** Summary of treatment machines and delivery modalities for SBRT audit plans.

Treatment machine	No. machines	Treatment technique	No. plans		
			Soft tissue	Spine	Lung
Conventional Linac—Varian	92	VMAT	243	212	221
Conventional Linac—Elekta	51	IMRT	6	19	3
Halcyon	9	3DCRT	4	−	8
CyberKnife	4	DCAT	19	2	27
TomoTherapy	3	Fixed Cone	3	2	1
		Iris Cone	3	2	2
		Helical VMAT	2	1	1
**Total**	**159**		**281**	**238**	**263**

**TABLE 3:** Summary of photon energy for SBRT audit plans.

	No. plans		
	Soft tissue	Spine	Lung
**6 MV**	60	60	72
**6 MV FFF**	129	112	133
**10 MV**	11	8	4
**10 MV FFF**	81	58	54
**Total**	**281**	**238**	**263**

**TABLE 4:** Summary of TPS and dose calculation algorithms for SBRT audit plans, as per AAPM TG329.^41^

Treatment planning system	Calculation algorithms	Dose reporting mode	Number of plans		
			Soft tissue	Spine	Lung
Eclipse	AAA	*D_w,w_*	41	46	24
	AXB	*D_m,m_*	131	83	130
Monaco	MC	*D_m,m_*	53	55	57
Pinnacle	CCC	*D_m,m_*	22	24	24
	AC	*D_m,m_*	7	7	10
RayStation	RS_CC	*D_w,w_*	16	11	11
	MC	*D_m,m_*	2	2	2
iPlan	XVMC	*D_m,m_*	2	3	2
Precision	Ray Trace	*D_w,w_*	4	2	–
	MC	*D_w,w_*	–	2	2
Tomotherapy Hi‐Art	CC	*D_m,m_*	3	3	1

The overall audit outcomes are given in Table 5. The overall pass rate was 96.4% for the soft tissue case, 90% for the spine, and 89.6% for the lung. Of the out of tolerance results, 91% were due to gamma pass rate < 90%, with the remaining 9% attributed to microDiamond point dose difference > 8%. Of the out‐of‐tolerance results, 44% were resolved with a repeat measurement, noting some facilities did not elect for a repeat measurement. Repeat audits were performed in the form of a repeat measurement of the original plan, after adjustment of machine parameters, or replanning of a case to improve plan quality.

**TABLE 5:** Summary of SBRT audit results for microDiamond point dose and Gafchromic film measurements.

		Film DTA @ 70% isodose						
Case	Film av. Gamma pass rate 5%/2mm (%)	Mean L–R (mm)	Mean A–P (mm)	Max PTV/Cord junction (mm)	microDiamond point dose % average	Pass (optimal level)	Pass (action level)	Out of tolerance
Soft Tissue	98.7 (3.7)	0.17 (± 0.62)	−0.28 (± 0.55)		−1.0 (± 2.3)	91.3%	5.1%	3.6%
Spine	96.5 (5.2)	−0.23 (± 0.60)		−0.21 (± 0.70)	0.1 (± 3.8)	75.0%	15.0%	10%
Lung	96.5 (6.5)		−0.21 (± 0.73)		−0.3 (± 3.2)	79.6%	10.0%	10.4%

*Note*: The average and standard deviation for each metric are shown.

**FIGURE 2:**
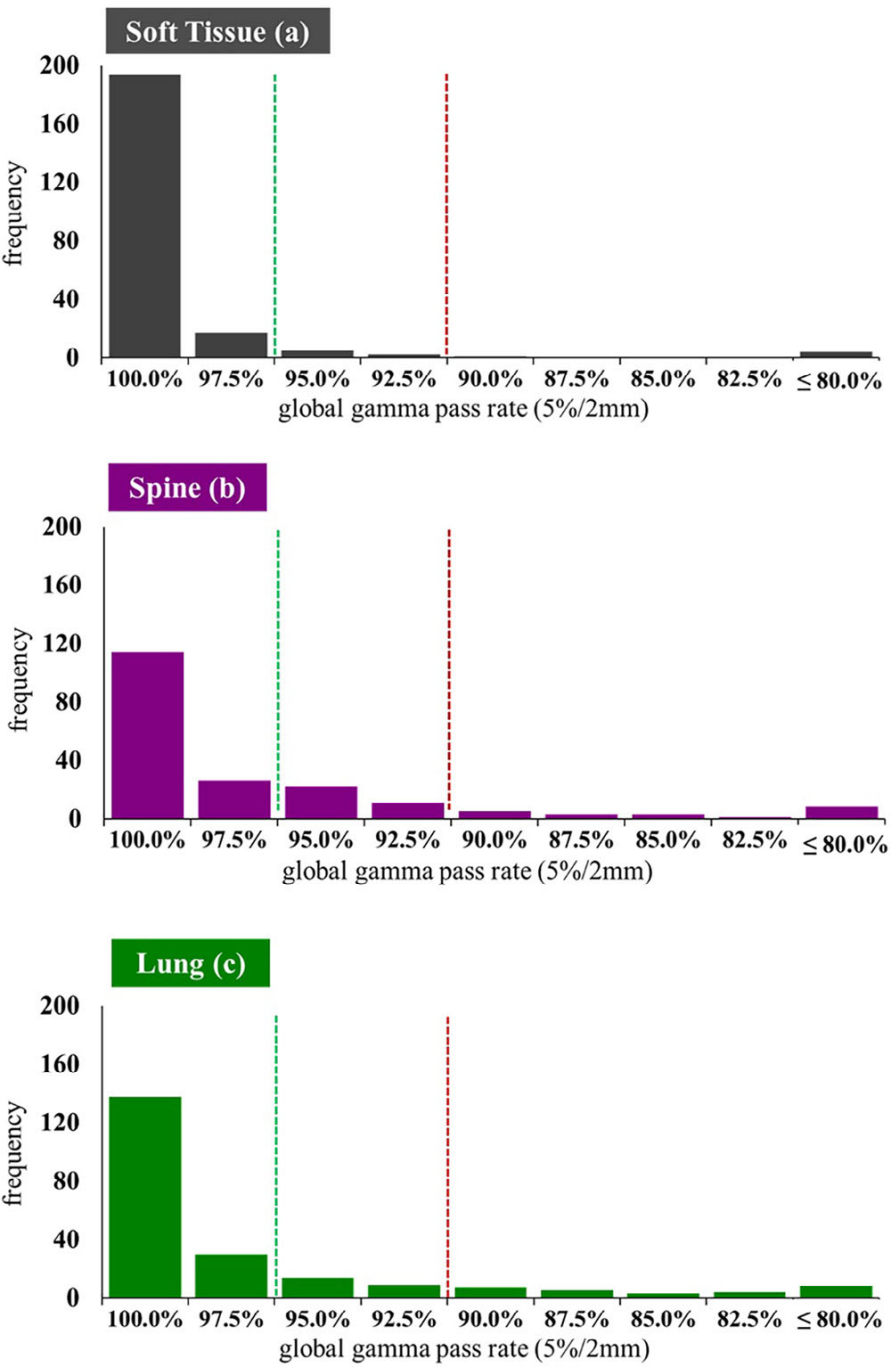
Overall audit results for SBRT dosimetry audit. Histograms show the number of audit cases returning a given gamma criteria pass range for the soft tissue (a), spine (b), and lung (c) cases, respectively. The dotted red lines indicate the out of tolerance thresholds for each metric, and the dotted green line indicates the pass (optimal level) threshold for the gamma metric.

Histograms of the gamma pass rates for each case are shown in Figure 2. The dotted lines indicate the action level (green) and out of tolerance (red) thresholds for each of the metrics.

**FIGURE 3:**
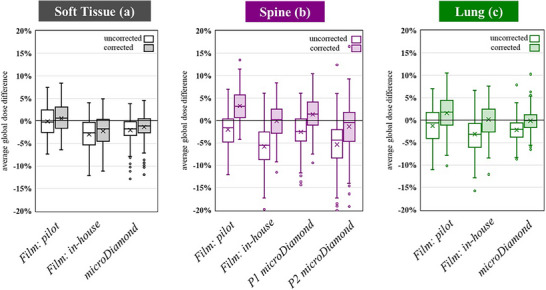
2D film average global dose variation and microDiamond point global dose variation in the PTV for soft tissue (a), spine (b), and lung (c). The corrected results have been tailored to the primary reporting mode of each TPS algorithm.

Whilst the gamma pass rate, microDiamond, and DTA metrics discussed were used to score the audit, dose differences provide better scope for trend analysis across the large number of audited plans. The average global dose differences in the PTV are shown in Figure 3 for each case. In this analysis, films analyzed using the pilot film method with commercial software FilmQA Pro are presented separately from the in‐house Matlab method. The PTV was defined as the area of the film bound by the prescription isodose for each case. The microDiamond point average global dose difference is also shown. Uncorrected and tailored medium corrected data are shown for each point of the analysis.

In general, the *k*_med_ corrected results showed improved agreement between plan and measurement, with all series showing statistically significant differences (*p* < 0.001) except the FQAP Soft Tissue (*p* = 0.237). The in‐house film method also generally showed statistically significant improvement in the audit results compared to the pilot method across all cases (Soft Tissue *p* < 0.001, Spine *p* < 0.001, Lung *p* = 0.22), suggesting some bias in results existed with the Pilot stage FQAP film dosimetry.

**FIGURE 4:**
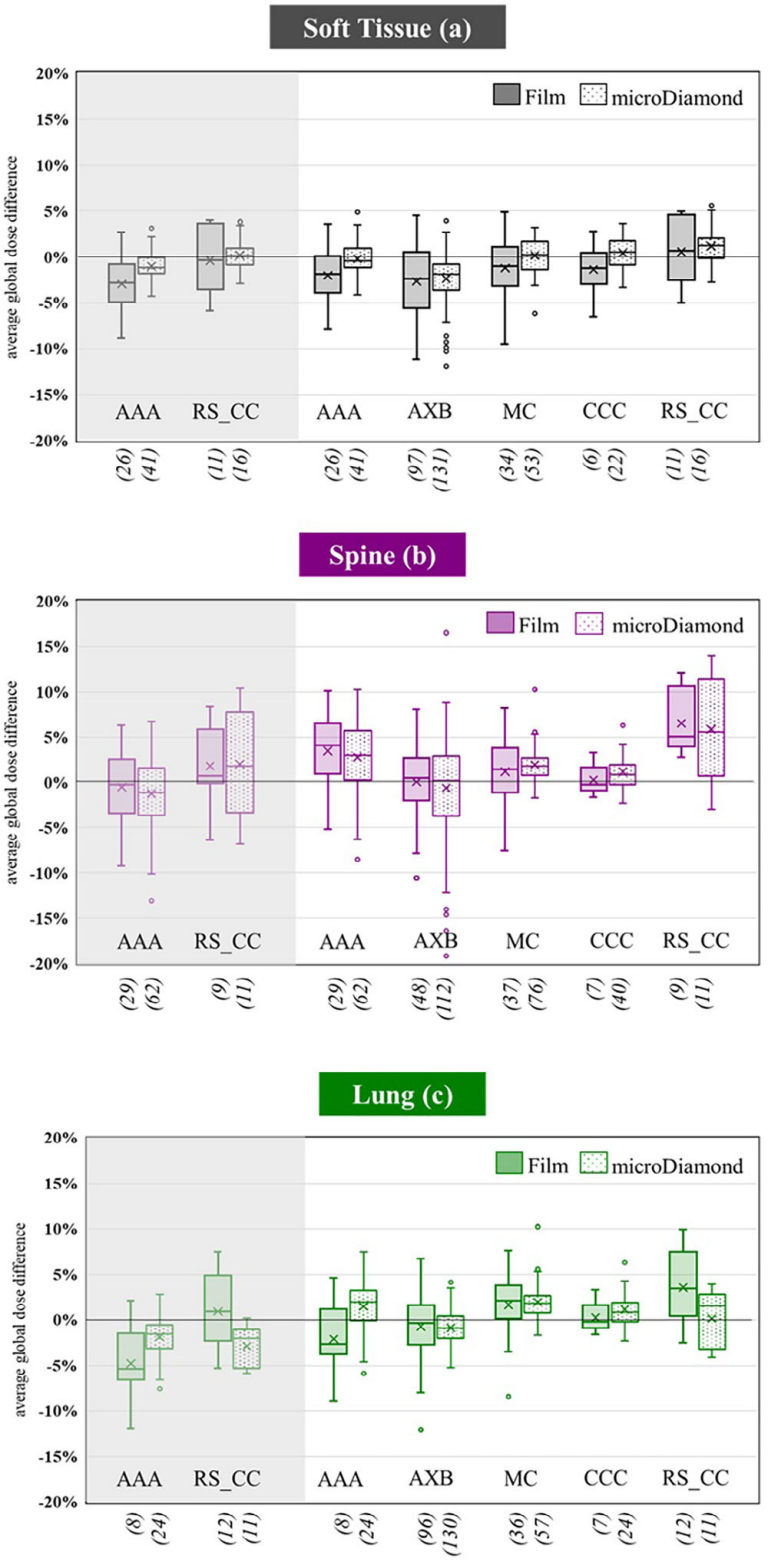
2D film average in PTV global dose difference and microDiamond global dose difference for soft tissue (a), spine (b), and lung (c). The results shaded in grey show the *D_w,w_* corrected results, tailored to the audit report. All other results are *D_m,m_* corrected.

A breakdown of the audit results by TPS algorithm is shown in Figure 4. Only algorithms with >5 plans are shown. In this analysis, the film processed using the pilot method has been omitted. For the *D_w,w_* algorithms, AAA and RayStation CC, the tailored *D_w,w_* corrected results are shown shaded in grey. For benchmarking the algorithms against each other, the results of all algorithms corrected according to *D_m,m_* are shown. The average global dose differences were <5% for all algorithms for both film and microDiamond measurements, except for RayStation CC. The film and microDiamond average global dose variation agreed to within 1% for the majority of algorithms, except in some cases of Pinnacle CCC and Raystation CC, which may be attributed to lower sample sizes.

**FIGURE 5:**
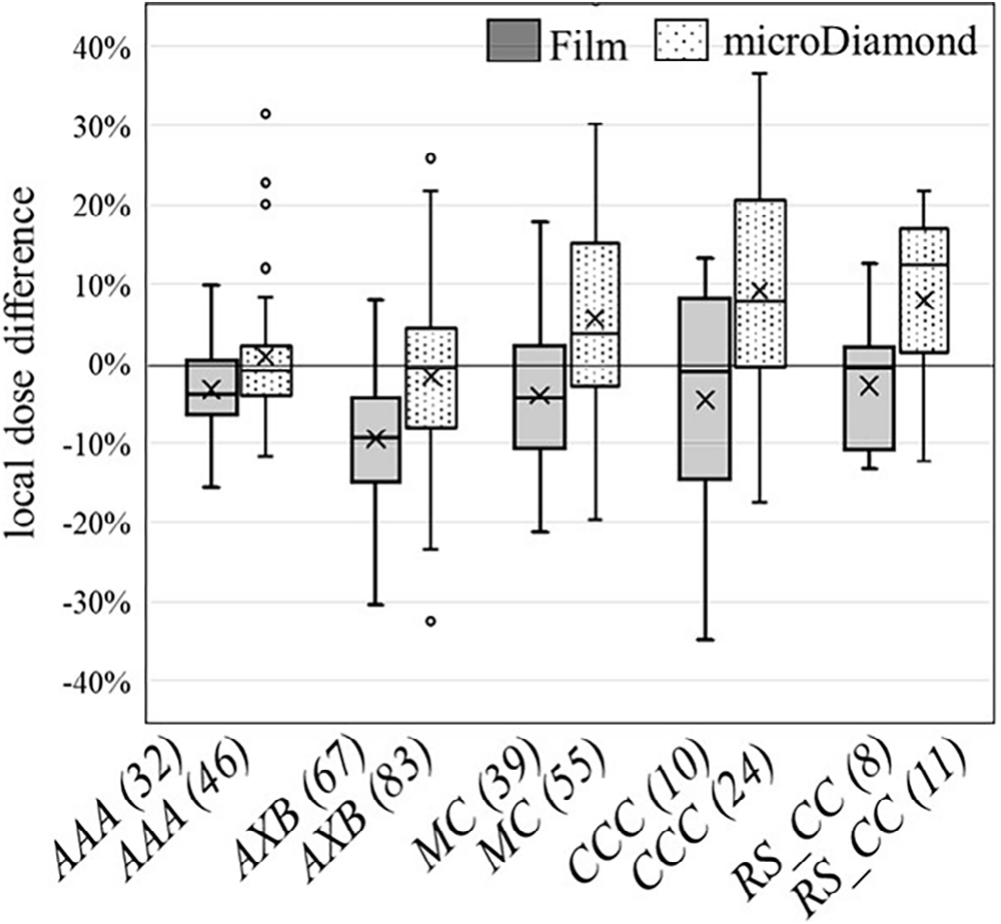
Film and microdiamond local dose differences in the spinal cord OAR. The film data corresponds to the average dose difference in a circular region of interest in the central 6 mm of the spinal cord structure.

Figure 5 shows the local dose variation in the spinal cord region for film and microDiamond. The film results show the mean difference in the ROI, taken as the pixels in the central 6 mm of the Spinal Cord structure, for the in‐house only film method. All measurements have been corrected as per *D_m,m_*. The largest discrepancy in the film measurements was 9.5% for AXB, which agrees with previously reported results.^43^ In the steep dose gradient spinal cord region, larger differences are seen between film and microDiamond measurements compared to the PTV measurements. The microDiamond data is more difficult to interpret due to the steep dose gradients and uncertainty in the placement of the interest point in the plan. The film, averaged over an ROI rather than a single point dose, may be a more reliable way to assess algorithm accuracy in the OAR region. In addition, there are measurement uncertainties that have not been addressed for the spinal cord microdiamond point. As discussed in Shaw et al.,^42^ additional corrections for small field sizes and perpendicular oriented detectors are required for microDiamond measurements in the SBRT audit. These corrections have been characterized for open fields and are therefore not appropriate to apply to the spinal cord point, which is measuring in a trough/out‐of‐field region. Characterization of such corrections was not performed for this study, as it was deemed to be of little value when the film provided both dose discrepancy and DTA information without requiring small field corrections.

In 96% of cases, a calculation grid spacing of ≤2 mm was utilized. No differences were seen in the audit pass rates between grid spacings ≥1 mm and ≤2 mm, and there was not enough data to assess if >2 mm made a significant difference to the audit result. In one audit plan, calculation on a 3 mm dose grid was determined to be a contributing factor to an out of tolerance result.

**FIGURE 6:**
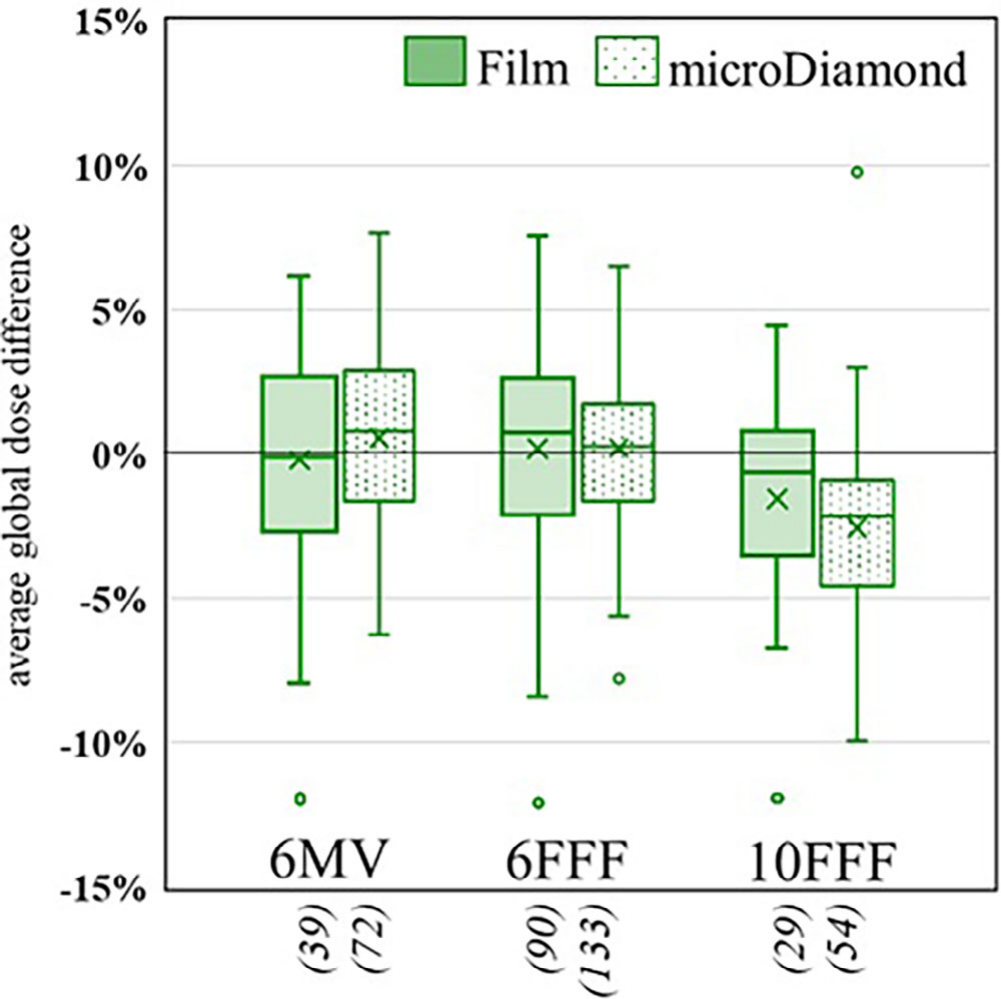
PTV average dose difference versus beam energy for the lung case.

The average PTV dose differences were within 1% for 6 MV, 6 MV FFF, and 10 MV FFF across soft tissue and spine. The 10 MV beam showed slightly larger differences (within 2.5%); however <10 plans were submitted for each case using this energy. As shown in Figure 6, small differences were seen in the 10 MV FFF measurements for both film and microdiamond compared to the 6 MV and 6 MV FFF results(film average 1.7% and microDiamond average 3.1%).

Out of the 782 plans measured, a total of 60 plans were out of tolerance (27 lung, 23 spine, and 10 soft tissue). Post audit analysis performed by the ACDS in conjunction with facility staff identified common failure modes, as shown in Table 6. In 44% of audit failures, a repeat measurement resolved the result to a pass outcome, although not all facilities elected to repeat the measurement. For IGRT failures, a re‐delivery of the original plan with correct image matching resolved the out of tolerance result in all cases. In other cases, re‐planning to improve plan quality (e.g., reducing modulation, removing a cold spot in PTV, or changing segmentation parameters) resulted in a pass outcome on repeat measurement. For one facility, realignment of the machine isocenter and redelivery of the original plan resulted in a pass outcome.

**TABLE 6:** Failure modes.

Failure mode	Soft tissue (*n*)	Spine (*n*)	Lung (*n*)	Total (*n*)	Total (%)
IGRT mismatch	4	5	13	22	36.7
In‐volume dose difference (low)	2	7	4	13	21.6
In‐volume dose difference (high)	2	3	7	12	20.0
Other shift/misalignment	1	5	0	6	10.0
Out of field dose (high)	1	2	2	5	8.3
Fusion of structure set to planning CT	0	0	1	1	1.7
Dose calculation grid size	0	1	0	1	1.7

## 4 DISCUSSION

The SBRT dosimetry audit provided a comprehensive assessment of a range of clinical practices, equipment, and challenging dosimetric conditions used in modern radiotherapy. High pass outcomes were seen across the three audit cases, demonstrating that SBRT has been implemented safely in the region. Given that one of the motivators for this audit design was for use in clinical trial credentialing, the results provide a high level of confidence in the delivered dose in SBRT clinical trials conducted during and subsequent to the period of results presented.^44^, ^45^, ^46^, ^47^ However, out of tolerance results were observed in a number of instances and could be attributed to dosimetric and positional delivery inaccuracies. Of the out of tolerance results seen in the ACDS audit, 44% of these cases were resolved with a repeat measurement, with initial failures attributed to an IGRT mismatch or machine isocenter misalignment. As expected, the simplistic soft tissue case performed the best, with an overall out of tolerance rate of 3.6% (10/281). The more complex spine and lung cases showed out of tolerance rates of 10% (23/238) and 10.3% (27/263), respectively. Whilst these out of tolerance rates are relatively high, they are comparable to the results seen by Imaging and Radiation Oncology Core (IROC), who found 15% and 22% failure rates for their spine and lung audits, respectively.^19^ Similar failure rates were also observed in the SBRT spine audit performed by Hardcastle et al.,^20^ where 11% of participating centers did not meet the established pass criteria. In SBRT lung audits conducted by the UK SABR group^14^ and the European Organization for Research and Treatment of Cancer (EORTC),^17^ pass or fail outcomes were not assigned to participating centers, however, a similar range of dose differences and gamma results were seen as in the ACDS audit. The ACDS lung audit did not include motion, which may explain the higher failure rate seen in the IROC cohort. The EORTC lung audit also observed poorer results in a dynamic lung target relative to a static target (92% vs. 71% average gamma pass rate for 3%/3 mm criteria respectively). These studies demonstrate the importance of including dynamic capability in further investigations by the ACDS.

Whilst gamma analysis is a widely accepted metric for SBRT QA, the criteria used for analysis varied widely between audit groups, and in facility PSQA. Whilst AAPM TG‐218 reports a universal tolerance limit for gamma criteria in IMRT and VMAT plans (3%/2 mm, 10% threshold and >95% points passing),^48^ no such guidelines exist for SBRT, and in any case, should be tailored to the resolution of the detectors.^49^ The ACDS SBRT audit was a multi‐institutional, true end‐to‐end audit including delivery by treating Radiation Therapists. Considering this, and the uncertainties in film dosimetry, we chose a gamma criterion of 5%/2 mm for analysis of film measurements. These tolerances were agreed upon in consultation with a Clinical Advisory Group of industry experts who assisted in defining thresholds for audit results and relating the metrics directly to patient safety. The tighter geometric component at 2 mm was chosen to reflect CTV‐PTV margin size and to highlight the clinical priority of geometric precision over dose difference in SBRT plans. The chosen dose difference metric at 5% considered the uncertainties in film measurement and was determined to be a clinically meaningful difference by the Clinical Advisory Group. The chosen gamma criteria may be wider than typically used in the more tightly controlled environment of an individual facility's PSQA that typically would not include IGRT treatment uncertainty. The IROC spine and lung irradiations used a single film gamma pass rate of 80% for 7%/5 mm global gamma criteria, and a dose difference of ±7% for TLD measurements. The ACDS audit pass criteria were stricter than the IROC pass criteria at 90% pass rate for 5%/2 mm global gamma criteria. The ACDS microDiamond point dose difference criteria was similar to the TLD variation limits in the IROC audits (8% vs. 7% respectively).^19^ The uncertainties of film measurement in the ACDS audit are on par with those reported by other groups. The UK SABR lung audit reported a dosimetric uncertainty of up to 7% for film (*k* = 2).^14^ Hardcastle et al.^20^ allowed up to 5% global dose scaling to account for the inherent uncertainties in radiochromic film, and the variation of scanner and film batch. Whilst other groups did not specifically report uncertainties, a range of global gamma criteria, local gamma criteria and point dose differences were used in the audit analyses.^14^, ^17^, ^20^ Although the scoring metrics should reflect the individual audit groups measurement techniques and uncertainties, as well as the clinical relevance of the audit cases, comparisons of large audit studies presents an opportunity for global harmonization on the most appropriate metrics for SBRT plan QA analysis.

Whilst the film served as the primary detector, post‐processing was required meaning the outcome of the case is not known on the day of the onsite visit. The microDiamond point dose served as a useful indicator of a potential delivery error in real time. A limitation of the ACDS audit was the use of a single piece of film, leading to an inability to assess spatial accuracy out of the film plane (superior/inferior for soft tissue and spine, anterior/posterior for lung). The microDiamond point dose result exceeding the ±8% threshold may indicate a positional offset in the direction of the film plane. Exceeding the threshold may also indicate very steep gradients within the dose distribution, which can assist in evaluating the robustness of the dose distribution. The DTA thresholds are also useful metrics for troubleshooting out of tolerance results and assessing the overall positional accuracy of the treatment. The DTA metrics used in this study are limited to 1D horizontal and vertical profiles at the center of the PTVs. This analysis could benefit from application of a 2D similarity metric such as Hausdorff distance,^50^ which could be the focus of further investigations for the ACDS.

The five most common TPS algorithms used in our region demonstrated acceptable results across the three audit cases for the majority of the audits conducted. As expected, larger discrepancies between planned and measured dose and a larger spread of results were observed in the spine and lung cases. This is likely due to the increased complexity of dose calculations due to the presence of inhomogeneities. The most accurate algorithms in the PTV region were MC, AXB, and CCC with median film and microdiamond results within 2% for all cases. AAA and CC performed similarly well in the soft tissue case but saw larger discrepancies in lung and spine. AAA on average underpredicted the dose in the lung by ∼3% and overpredicted the spine dose by 3%. The difference in lung has been previously reported in previous analysis by Shaw et al.^25^ Although the Pinnacle CCC performed well, the CC implementation in RayStation TPS displayed larger differences in spine overpredicting by 5%. There was limited data with only 14 spine plans tested for CC, and this result may be biased by IGRT or other errors.

A previous study by Hughes et al.^43^ of the ACDS SBRT spine audit dataset showed an underestimation of dose in the spinal cord region by the TPS when calculating with AXB and MC algorithms. This was not reflected in the overall audit pass rate for the spine case in this study, as the number of pixels in the Spinal Cord that did not meet the gamma criteria was not sufficient to make the overall percentage of points passing <90%. In the OAR spinal cord region, AAA and MC performed well as determined by film measurements with median local differences <4%. In both the CCC and CC film and microDiamond results, larger spread of data was observed with lower plan numbers. The underprediction of dose in the Spinal Cord OAR region by AXB as discussed by Hughes et al.,^43^ was observed in the film results (10% median local difference) but not in the microDiamond. In the previous study, CC13 ionization chambers and the PTW Octavius 2D ionization chamber array were used to measure the dose to a C‐shaped target. Whilst the geometries of the C‐Shape are different from the SBRT spine case, the same “wrap around” target volume with adjacent OAR is employed. The SBRT film results presented in this study agree with the previously reported results with other detectors, leading the authors to distrust the microDiamond measurements. There are a number of reasons for this unreliability; the microDiamond measurement in this scenario is only a single point and may be subject to large uncertainty due to positional offsets, steep dose gradients, and uncertainty in the placement of the interest point in the plan. In addition, corrections for small fields and perpendicular detector placement have not been characterized in a dose trough, and would offer little value when there is a reliable detector in film which offers both dose and positional uncertainty.

Anecdotally, there is some hesitation amongst clinical Medical Physicists when using 10 MV or 10 MV FFF in lung SBRT due to lateral electron disequilibrium for small fields in lung, and longer range of secondary electrons in higher energy beams.^51^, ^52^ Despite these traditional views, several participating centers elected to use 10 MV FFF beams in the audit plans, presumably for the dose rate benefits if the patient is required to be in breath hold. The results of this study indicate only small differences with 10MV FFF compared to 6MV/6MV FFF (1.7% for film and 3.1% for microDiamond), which are within the audit tolerance and should be carefully considered against the dose rate benefits of the higher energy beam and the potential to reduce patient intrafraction motion.

The SBRT protocol also did not prescribe the facility QA method or metrics used for analysis, and only required the plan to pass internal QA prior to delivery in the audit. As a result, a wide range of PQSA techniques, equipment, and metrics were observed across the facilities participating in the audit. As the ACDS only audited plans which had passed facility PSQA, this was not an indicator of audit result, highlighting the need for facilities to undergo independent dosimetry audits. This is in agreement with the analysis performed by Kry et al.,^53^ where the facility IMRT QA lacked sensitivity to detect the suboptimal results seen in the IROC Head and Neck phantom audit irradiations.

Whilst the audit can be considered an end‐to‐end test, a noticeable aspect that was missing was the physician's definition of SBRT treatment volumes. The SBRT phantom was equipped with easily discernible structures, unlike patients whose anatomy is more open to interpretation. In the SBRT audit, the PTVs were defined from physical targets in the phantom, with margins adapted from clinical trial protocols.^35^, ^36^, ^38^ Although inter‐observer contouring presents a large source of uncertainty in SBRT,^54^, ^55^ comparison is outside the scope of this work and could be considered during credentialing for individual clinical trials.

## 5 CONCLUSION

The ACDS has developed a comprehensive SBRT dosimetry audit across a range of clinical cases and delivery systems. High pass rates were seen across soft tissue, spine, and lung cases, indicating the safe implementation of SBRT practice in Australia and New Zealand. Suboptimal results observed in spine and lung cases can be attributed to differences in dose calculation algorithms, dosimetric offsets, and IGRT mismatches. Facility PSQA results were not an indicator of audit outcome, highlighting the importance of treating facilities to participate in independent dosimetry audits. Dynamic capability should be prioritized in future developments to evaluate the accuracy of clinical motion management strategies.

## CONFLICT OF INTEREST STATEMENT

The authors declare no conflicts of interest.
